# A Framework for Introducing Global Health Innovations to the US

**DOI:** 10.5334/aogh.3600

**Published:** 2022-08-08

**Authors:** Soo Yun Shin, Maria Knight Lapinski, Syed Ali Hussain, Yvens Rumbold, Ruth Osoro, Donald Shell, James W. Dearing

**Affiliations:** 1 Department of Communication, Seoul National University, Seoul, KR; 2 Department of Communication, Michigan State University, East Lansing, MI, US; 3 Health & Risk Communication Center, Michigan State University, East Lansing, MI, US; 4 Walter Cronkite School of Journalism and Mass Communication, Arizona State University, Phoenix, AZ, US; 5 Department of Communication Arts and Sciences, Pennsylvania State University, University Park, PA, US

**Keywords:** health innovation, global health, diffusion of innovation, reverse diffusion, designing for diffusion

## Abstract

**Background:**

Across the globe, there are successful health innovations that could help improve public health in US communities at lower cost and with higher effectiveness than standard practice. However, which factors should be considered to heighten the likelihood of successful transfer of global health ideas to the US still warrants more empirical investigation.

**Objective:**

This study aimed to develop a conceptual framework delineating important factors to be considered for successful introduction of global health innovations to US communities, based on diffusion of innovations literature and case studies of global health innovations that have been adopted in US communities.

**Methods:**

Five global health innovations adopted in US communities were selected based on expert panel recommendations and a review of academic and gray literatures. These innovations had diverse origins (Columbia, Mexico, South Africa, Sweden, and Wales) and exhibited various means of achieving desired health outcomes. We conducted archival research and 27 interviews (42 interviewees) with leaders and stakeholders of the five innovations to identify important factors for the transfer of global health innovations to the US.

**Findings:**

Six factors were determined to be important for global health innovation adoption in the US: (1) innovation attributes, (2) linking agents, (3) inter-organizational partnerships, (4) scale up strategies, (5) implementation processes and outcomes in US communities, and (6) policy and social context. These factors correspond well to factors emphasized in the diffusion of innovation literature, although the importance of some sub-factors (e.g., stigma regarding the origin of innovations) diverged from the literature.

**Conclusions:**

Based on our findings, we developed the Designing for Diffusion Framework for Global Health Innovations. The framework provides a comprehensive picture of factors that can be facilitators or hindrances for moving a global health innovation to the US to help smooth the diffusion process for better adoption and implementation in US communities.

## Introduction

For many years, studies of global health practices and programs have reported on the diffusion of these innovations from high-income countries to middle- and low-income countries [1]. More recently, health system experts in high-income countries have suggested looking abroad for effective solutions to health disparities, middling quality and high cost [2,3,4]. US-based healthcare providers such as Henry Ford Health System have founded global health units to seek and transfer promising practices from abroad [5]. Nonprofits such as the Institute for Healthcare Improvement and Global to Local have launched efforts to bring international solutions to US higher education institutions such as Duke University that have created emphases on the transfer of global health interventions to US communities. Philanthropies in the US such as the Commonwealth Fund and the Robert Wood Johnson Foundation also have programs focusing on moving global ideas to the US [6]. Online clearinghouses exist that identify, assess and categorize thousands of innovations from throughout the world that may be candidates for diffusion [7].

There are known barriers to the adoption and spread in the US of health innovations from other countries [8]. There are also some unknowns such as whether barriers are greater for innovations from other high-income countries or those from middle- or low-income countries. It may be the case that innovations coming from high-income countries face fewer barriers to diffusion in the US because of similar sets of circumstances and constraints. Or perhaps innovations that thrive and survive in low-resource countries have demonstrated great resilience and thus are well-positioned to perform well in high-resource countries.

Explanations for innovation diffusion or lack thereof have regularly appeared, such as an assessment of the rapid spread of Tobacco 21 policies in the US [9] and comparison of the diffusion of the Drug Abuse Resistance Education program with diffusion of syringe needle exchange programs [10]. These assessments generally use three well-established sets of factors that typically do a good job of accounting for why a health innovation does or does not spread: First, the attributes of innovations such as perceived costs, compatibility with conditions at adopting sites, and degree of complexity; second, the characteristics of the adopting social system and the members to which an innovation will be communicated; and third, the socio-environmental context at the time that an innovation is communicated to social system members [11,12,13]. Our main research question was: Are the factors that explain global-to-US diffusion different than those specified in the general diffusion model? Perhaps the set of contributing factors or their importance differs [14].

We aimed to develop a conceptual framework to guide the importation and adaptation of global health innovations into US contexts. We first conducted a literature review to identify key factors that traditionally account for diffusion as well as additional factors that could be important for the diffusion of global ideas in the US. The identified factors included (1) the qualities of an innovation itself such as perceived costs, effectiveness, external validity, compatibility, simplicity, trialability, observability and stigma [15,16,17], (2) the role of key players and linking agents such as policy entrepreneurs, knowledge brokers, innovators and organizations such as NGOs, government agencies and research institutes [18,19,20], (3) the formation and operation of inter-organizational partnerships at the national level and community level [21,22], (4) different scale-up “pathways” such as branching, affiliation, distribution networks, or dissemination, the inclusion of a choice of innovations, and the inclusion of a delimited set of alternatives to how an innovation could be implemented [23,24], (5) whether multiple layers of decisions are required in an adopting community, monitoring for implementation fidelity, adaptation strategies for sustained use, obtaining process and outcome data for effectiveness verification [1,25,26], and (6) external policy, economic and social environments [27,28].

By selecting five cases of the import of global health innovations to the US and conducting in-depth interviews with key actors for each innovation, we investigated whether the factors identified in previous literature helped explain successful and unsuccessful global health innovation diffusion to the US. Then, drawing upon the case study results, we propose a *Designing for Diffusion (D4D) Framework for Global Health Innovations*.

## Methods

### Case Identification

Innovations were identified and selected through multiple steps. A database of candidate innovations was created through examination of the published and gray literature and web searches. Our initial search focused on pro-social public or environmental health practices and programs that originated outside the US and had spread to at least one other country that was not the US. We searched academic journals (e.g., *JAMA, PLoS One*), practitioner magazines (e.g., *MIT Innovations* and *Stanford Social Innovation Review*), websites (e.g., Ashoka and Acumen Foundation), and materials from aid organizations (e.g., World Bank and USAID). Next, we met with the Robert Wood Johnson Foundation (RWJF) Global Ideas for US Solutions team for suggestions of candidate innovations. We also convened a project advisory group of experts in healthcare and public health for their suggestions of candidate innovations and possible interviewees. These steps yielded 39 innovations.

**Figure 1:**
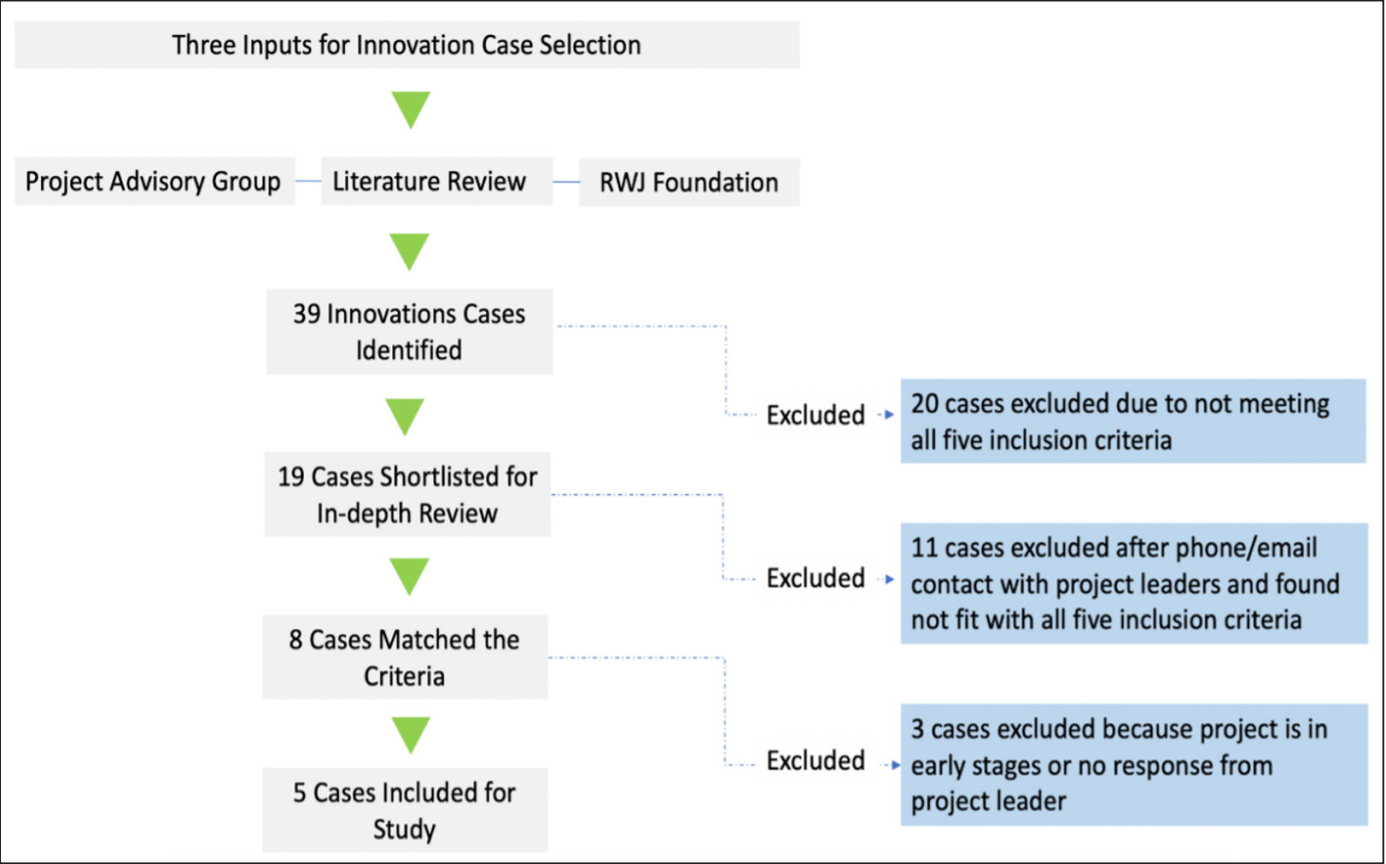
Process for selecting candidate cases of global health innovations.

Among the 39 innovations, we excluded 20 innovations that did not meet our five inclusion criteria: The innovation should (1) be of international origin with spread to at least one other country other than the US, (2) be pro-social, (3) be from a low- or middle-income country or serve low-income people (4) have spread to the US, and (5) have process or outcome data to determine or at least suggest effectiveness of the innovation. We then contacted representatives of the 19 remaining innovations to assess whether they were still in operation and gauge their willingness to answer questions about their experiences. This last step reduced the set to five innovations (Figure 1).

The five global health innovations we selected are Ciclovía (originating from Colombia), ConsejoSano (Mexico), Cardiff Violence Prevention Model (Wales), the Swedish Rheumatology Quality Registry (Sweden), and AgeWell Global (South Africa). Below we provide a summary regarding each innovation.

1. **Ciclovía.** Originating in Columbia and having been adopted by many US communities including Los Angeles, New Brunswick, Wayne County Michigan and Portland, Oregon, Ciclovía is a physical activity promotion and social isolation reduction program in which streets are closed temporarily to automobiles for the benefit of cyclists, runners, and pedestrians.
2. **ConsejoSano.** Originating in Mexico and having scaled up in parts of California, Texas, Illinois and New York, ConsejoSano is a private company that contracts with health insurers and community clinics in the US to help clinics convince low-income community members whose native language is not English to come to the clinics for health services.
3. **Cardiff Violence Prevention Model.** Originating in the United Kingdom and with implementations in Atlanta, Decatur, and Milwaukee, the Cardiff Violence Prevention Model provides a way for communities to gain more information about where violence occurs, and when and how to prevent it by forming partnerships between hospitals, law enforcement, and community groups such as local bar owners.
4. **Swedish Rheumatology Quality Registry.** Originating in Sweden and in the US having been reinvented as the Swedish Quality Registry at Dartmouth College, this innovation enables both patients as well as health care providers to input information about a patient’s progress in care, for patients with cystic fibrosis, inflammatory bowel disease, and other conditions through partnerships with disease-specific national foundations and associations.
5. **AgeWell Global.** Originating in South Africa and with pilot implementations in Cleveland, Fort Lauderdale, and New York City, AgeWell Global is a model of elder care coordination combining peer-based social engagement and mobile technology to improve health outcomes for elders and reduce medical costs for healthcare organizations.

### Interview Procedure

We conducted telephone and video interviews about the five innovations with founders, international leaders, policy makers and researchers. We then scheduled and conducted site visits to interview implementers across the US. In total, we conducted 27 interviews with 42 interviewees during 2018. Each interview lasted between 1–2 hours involving at least two study team members. A semi-structured interview protocol was used, and all interviews, except one, were digitally recorded and manually transcribed. All procedures were approved by the Michigan State University Institutional Review Board (Ref. No. STUDY00000849).

Interviewees were read or shown a consent statement and verbally consented to participate with digital recording. After some rapport-building questions, interviewees were asked about key factors identified in the literature review in relation to their innovation diffusion to the US: the innovation itself (innovation attributes), key individuals and roles they played in spreading the innovation (linking agents), key organizations important to moving the innovation to the US (inter-organizational partnerships), the approach taken to spread and scale up (pathways and scale up strategies), experience with the US communities (receiving US communities and organizations), and other external factors (context).

### Data Analysis

While we used prior research to draft a set of key factors for global health innovation diffusion to the US to add rigor to our case study results, we used an inductive coding approach to analyze the data by which we would assess the pre-identified factors. Specifically, thematic analysis was used for identifying, coding and making sense of patterns within the interview data without a priori categorization [29]. After transcribing the interview recordings, we had a total of 436 single-spaced pages of text to analyze. Three researchers each reviewed three randomly selected transcripts out of the 27 interviews to identify themes and subthemes that were common within the data. The themes generated in this portion of the analysis were not constrained by particular interview questions but were developed on the basis or responses given to multiple, related interview questions. Next, a codebook was developed based on the captured themes, which was used as the basis for the second round of review for all the transcripts, also allowing for additional themes to emerge. The three researchers analyzed nine transcripts each and all data were identified as connected to a theme or subtheme or else as irrelevant to the themes. A fourth researcher reviewed the raw transcripts as well as the thematically coded transcripts as a validity check. Lastly, the major themes and subthemes and their relationships to the key factors were discussed and determined by the team members.

## Results

### Global Innovation Attributes

For all five of the global innovations we studied, we found a consistent appreciation for the importance of (1) a positive benefit/cost assessment, and (2) compatibility of the innovation with the adopting US community context. Interviewees talked about these attributes as essential to the diffusion and scale up challenge. Indeed, their characterization of these attributes is almost a given, or an assumption, that an innovation must satisfy these requirements in order to have a chance at being adopted and implemented by others. For example, several people with Cardiff indicated that there is little financial incentive for hospitals to adopt the innovation, yet the public health *benefits* of identifying and reducing violence were discussed by all participants as being very important and *compatible* with the values of people within the organizations connected to the innovation. Similarly, Ciclovía was seen as *beneficial* and *compatible* with peoples’ desire to reduce motorized transport use and increase physical activity in their city, their value on environmental sustainability, and a desire to ride bikes to “reclaim” city streets from cars. However, the perceived costs, such as financial costs borne by the organizing entity to hold the event, sustain the necessary staffing, leadership, or volunteers, and potential costs to businesses in lost revenue due to customers being unable to drive to or park, were described as major hindrances for adopting and implementing the innovation by the interviewees.

Other attributes mentioned regularly by interviewees include (3) the importance of simplicity, (4) trialability, and (5) observability. For example, the initial ConsejoSano service was *simple* to use as it “allowed anyone in the US to tap their phone and within 10 seconds they were talking to a native Spanish-speaking doctor,” said one leader, “24 hours a day.” A newer version of ConsejoSano with text messaging was perceived to be similarly easy to use. The Swedish Quality Registry was evaluated highly in terms of trialability. The registry was designed to be “co-created” and as such, *trialable* by stakeholders and potential users prior to implementation. For example, one participant from the Dartmouth group said: “We can test and refine, test and refine before we go to the next person and be like, ‘try this.’ We can do small tests of change and learn quickly, really quickly.” Ciclovía interviewees considered *observability* to be another major explanation for its spread. Ciclovía is highly visible and experiential; thousands of people out walking and biking and skating in the street, often talking and sightseeing, in multigenerational fashion. Many interviewees said that they were inspired to start or get involved in a Ciclovía in their city of residence after attending or seeing a Ciclovía in another city.

External validity and stigma regarding the origin of innovations were not mentioned as important factors for successful adoption in the US For Ciclovía and ConsejoSano, the foreign names of the innovations even worked toward their advantage for diverse populations. A Ciclovía interviewee said, “I think people just look at what it is, and if it means something to them, I don’t think it matters where it comes from. It looks like fun, so they go for it.” It was also clear from our results that for some residents, close identification of an innovation with a low-income country was a positive attribute, not a negative attribute.

### Linking Agents

For all five innovations, linking agents played a key role in the movement of innovations across geographic locations. Most innovations moved to the US through organizational linkages, although some individuals played the key role in the process, including: Researchers affiliated with universities (Cardiff Violence Prevention Model, Swedish Quality Registry); city officials (Ciclovía); funders with contacts internationally as well as domestically (Cardiff Violence Prevention Model Swedish Quality Registry, AgeWell); or private companies that brokered relationships with healthcare organizations (ConsejoSano). For instance, movement of the Cardiff Violence Prevention Model to the US was initiated by a contract between two researchers. Publication of the first results about the Cardiff Model in an academic journal by a professor at Cardiff University led to an inquiry from a professor at the University of Pennsylvania. That professor in Philadelphia communicated with a former student who worked at the Robert Wood Johnson Foundation, who brought news of the Cardiff Model to the foundation. This information led to a grant. In the case of Ciclovía, initial linkage was accomplished by members of the international biking community and officials in various US cities. Key individuals also functioned as linking agents for ConsejoSano, as an interviewee said, “He (…) was born in Mexico and immigrated to the US and has been living in the San Diego area for 30+ years. He is the Chief Medical Officer of an FQHC [Federally Qualified Health Center]. He was introduced to us through our president and became a champion and supporter.”

### Partnerships

For these five innovations, a mix of community, national, and international partnerships were found. Partnerships local to US communities were the basis for implementation in organizations and in communities. National and international partnerships allowed for wider adoption by more sites. For example, AgeWell established pilot demonstrations of how it could effectively work locally by forming partnerships with community organizations serving seniors and with health care delivery systems in those communities, such as Henry Street Settlement in New York and Fair Health Partners in Cleveland. The Swedish Quality Registry involved local, national and transnational partnerships. Local partnerships include patient groups, clinical care teams and IT departments in healthcare systems. National actors include funding agencies, registry advocacy organizations and research organizations. The original transnational tie between the Karolinska Institute and Dartmouth College is maintained through continued research collaboration. In the case of Ciclovía, the nature and complexity the partnerships varied by community, with large cities hosting complex events that require professional event management and the central involvement of many municipal units. A leader in Los Angeles said: “Part of the process was really making sure that the other departments, including transportation, sanitation, sewage services, parks & rec, and you can just keep going down the list, were all on the same page.”

### Scale Up Strategies

The cases we studied commonly relied on the dissemination of information to generate interest among potential adopters about their innovation. In particular, Ciclovía exclusively relied on word-of-mouth as a means of scaling up. Compared to Ciclovía, the other four innovations used multiple scale up pathways in addition to dissemination of information. For example, AgeWell and Cardiff Model followed a strategy of scaling by finding and training affiliates in each new US community. The Swedish Quality Registry and ConsejoSano found distribution network organizations that already had access to health care providers and patient populations. ConsejoSano also relied on reinvention extensively to be compatible with consumer and health system parameters in the US market, which helped scaling up their service. Specifically, they reinvented (1) the services they provide, from answering questions from individuals about health and disease to convincing under-served individuals to come into a nearby clinic, (2) the means of communication, from telephone conversations with doctors to text messages with ConsejoSano staff, and (3) who takes the initiative, from an in-reach or demand-based model of service provision to an outreach or supply-based model.

### US Adopting Communities

Adoption decisions, for the cases we studied, were generally made in a collaborative fashion with organizational partners at multiple levels. For example, the adoption of the Cardiff Model in the US was facilitated by staff at the Robert Wood Johnson Foundation and the Centers for Disease Control and Prevention, and those staff acted as intermediaries to US communities through personal contacts. Implementation, especially as the global ideas moved across sites, involved adaptation while maintaining a core identity. For instance, for the Cardiff Model implementation in Milwaukee, nurses rather than front office staff collect incident data from victims, and a university-based injury center receives the hospital intake data and cleans, analyzes, and maps those data rather than the police department having this responsibility, unlike the original concept of the innovation. Delineating a core identity for the innovation was typically done by the inventor who communicated that identity as the innovation diffused. Sustained use of the innovation was dependent on the will of particular people or groups of people as well as availability of financial and other resources. The gathering and communication of evaluation data often helped make the case for sustainability, but positive outcome data were not sufficient to ensure the long-term continuation of an innovation. For example, even though AgeWell in Florida had evidence for its success—reducing hospital readmissions by 46% over 90 days post discharge—the program could not continue due to an earlier commitment by a leader and staff in the partner hospital to an alternative community health worker program for seniors, which reduced the focused effort and resources for AgeWell.

### Context

The macro-policy context functioned in an enabling role in the cases we examined. The historical context and the timing of the introduction of global innovations played both a hindering and facilitating role in several of the cases. For example, the large and growing Spanish-speaking populations in the US, along with the Affordable Care Act, provided a ready and underserved market for ConsejoSano, which was able to use digital and mobile health technology to serve the target population at low cost. In the case of the Cardiff Violence Prevention Model, it diffused in places where, at the time of adoption, violence was a priority for the community since the rates of intentional injuries had increased and were being covered by local mass media. The Cardiff Model thus became a solution to a newly important and prioritized problem. The built and natural environment was another contextual factor contributing to the adoption and sustained use of Ciclovía—temperate weather for being outdoors, conducive city spaces, and supportive infrastructure of biking/non-motorized transport. The litigious nature of US society in the event of a Ciclovía being targeted for an attack or an accident occurring was also mentioned as a contextual factor that needs to be addressed when presenting the Open Streets idea to city officials.

## Framework Development

### The Designing for Diffusion (D4D) Framework for Global Health Innovations

**Figure 2:**
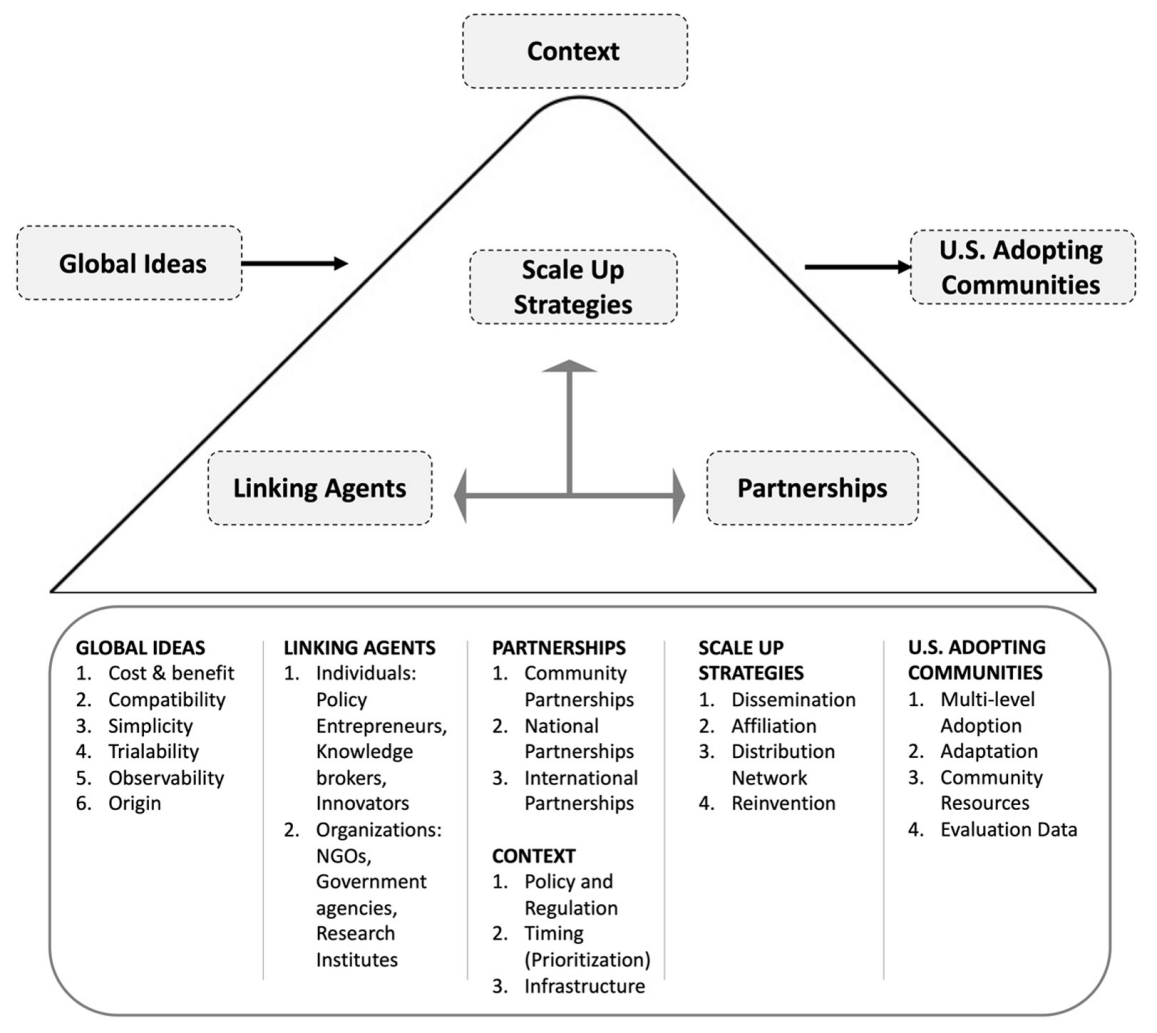
Designing for Diffusion (D4D) Framework for Global Health Innovations.

Our purpose was to specify a conceptual framework that could account for why global health innovations do and do not diffuse in the US. Through literature review and five case studies with in-depth interviews with innovation leaders, we were able to identify the importance of (1) attributes of global innovation ideas, (2) linking agents, (3) inter-organizational partnerships, (4) scaling strategies, (5) actual implementation process and outcome in US adopting communities, and (6) broader contexts. Although the importance of sub-components of the six main factors can vary across innovations, the six factors were consistently mentioned by our interviewees as determinants for successful diffusion to the US. Our framework is presented in Figure 2.

It is worth noting that some sub-components identified in interviews differed from those identified in the literature review process, which led us to revise the framework accordingly. For example, we had anticipated that *stigma* would be a problem for these innovations [16,17], especially those from low- and middle-income countries. We found just the opposite. Some interviewees said that the fact that an innovation had come from a low-resource country was appealing to people in their organization and the communities. Our interviewees suggested that multiculturalism and low-resource settings, as exciting and exotic sources of innovative ideas, were appreciated in their communities. We conclude that this sub-component should be reconceptualized as *origin*, since our results suggest that innovation origin can be viewed as a positive attribute. In addition, we added *international partnerships* for inter-organizational partnerships as we found more cases where the original innovators or promoters of the global ideas have consistently worked across nations even after the initial role of linking and introducing the idea to the potential US adopting decision makers is over. The international partnerships played a significant role in preserving the key element of the innovation, while making necessary adaptations to the US communities over the adoption process. Also, sub-components of scale-up strategies were narrowed down by removing the ones that were not mentioned by interviewees to be of importance. For instance, either providing a choice of innovations by jointly offering the target innovation with other innovations or providing or receiving implementation alternatives were not mentioned as scale-up strategies and thus were removed from the framework.

In sum, our framework suggests that (1) *global ideas* with certain characteristics have a higher chance of being picked up by (2) *linking agents* who seek and build (3) *partnerships* to initiate the adoption process. Then the linking agents and partnerships select (4) *scale up strategies* to promote in (5) various *US adopting communities*. Meanwhile, the (6) social *context* at the time also affects how easily the above processes are performed. When designing for the diffusion of innovations from abroad to the US, consideration of these six factors, as well as how they interact, can improve the likelihood of successful adoption and implementation.

## Conclusions

Novel and effective health practices and programs exist all over the world. Finding ways to move those ideas—diffusing those innovations either in their original form or more commonly suggesting how certain adaptations can smooth their adoption and implementation into new countries and their communities—is important translational work. Based on an extensive literature review regarding the diffusion of innovations and a series of in-depth interviews about five global health innovations that we studied as cases of global-to-local diffusion, we developed a *Designing for Diffusion Framework for Global Health Innovations*. We believe this framework provides guidance about factors to consider when attempting to diffuse global health innovations, especially to US communities from low- and middle-income countries, which characterized the five innovations we studied. The D4D framework can be used by public health decision makers to identify promising innovations to import into their own communities by considering the innovation’s attributes, such as cost or compatibility. The framework can also help public health stakeholders to intentionally reinvent innovations prior to launching a diffusion or dissemination effort, as well as suggest the ways that an innovation may benefit from adaptations made by an adopting community. For example, the a priori identification of key linking agents who can access or initiate international partnerships or local community partnerships for effective implementation are clearly important tasks.

As next steps, we hope that the present framework can be examined and further tested by others with new cases of global health innovations, preferably that are at the discussion stage. Tracing the spread of a new global innovation from its dissemination of information to demonstrations of it, adoption by receiving communities, changes made during local implementation, and then sustained enactment and the occurrence or not of second-generation diffusion to yet other sites would likely provide additional evidence to better specify the D4D framework for transnational diffusion.
